# Volume Guarantee High-Frequency Oscillatory Ventilation in Preterm Infants With RDS: Tidal Volume and DCO_2_ Levels for Optimal Ventilation Using Open-Lung Strategies

**DOI:** 10.3389/fped.2020.00105

**Published:** 2020-03-24

**Authors:** Funda Tuzun, Burak Deliloglu, Merve Meryem Cengiz, Burcin Iscan, Nuray Duman, Hasan Ozkan

**Affiliations:** ^1^Division of Neonatology, Department of Pediatrics, Dokuz Eylul University Faculty of Medicine, Izmir, Turkey; ^2^Division of Neonatology, Department of Pediatrics, Tinaztepe University Faculty of Medicine, Izmir, Turkey

**Keywords:** HFOV, volume guarantee, lung recruitment, VThf, DCO_2_, frequency, RDS, lung-protective strategies

## Abstract

High frequency oscillatory ventilation with volume-guarantee (HFOV-VG) is a promising lung protective ventilator mode for the treatment of respiratory failure in newborns. However, indicators of optimal ventilation during HFOV-VG mode are not identified yet. In this study, we aimed to evaluate optimal high-frequency tidal volume (VThf) and the dissociation coefficient of CO_2_ (DCO_2_) levels to achieve normocapnia during HFOV-VG after lung recruitment in very low birthweight infants with respiratory distress syndrome (RDS). Preterm babies under the 32nd postmenstrual week with severe RDS that received HFOV-VG using open-lung strategy between January 2014 and January 2019 were retrospectively evaluated. All included patients were treated with the Dräger Babylog VN500 ventilator in the HFOV-VG mode. In total, 53 infants with a mean gestational age of 26.8 ± 2.3 weeks were evaluated. HFOV mean optimal airway pressure (MAPhf) level after lung recruitment was found to be 10.2 ± 1.7 mbar. Overall, the mean applied VThf per kg was 1.64 ± 0.25 mL/kg in the study sample. To provide normocapnia, the mean VThf was 1.61 ± 0.25 mL/kg and the mean DCO_2_corr was 29.84 ± 7.88 [mL/kg]^2^/s. No significant correlation was found between pCO_2_ levels with VThf (per kg) or DCO_2_corr levels. VThf levels to maintain normocarbia were significantly lower with 12 Hz frequency compared to 10 Hz frequency (1.50 ± 0.24 vs. 1.65 ± 0.25 mL/ kg, *p* < 0.001, respectively). A weak but significant positive correlation was found between mean airway pressure (MAPhf) and VThf levels. To our knowledge, this is the largest study to evaluate the optimal HFOV-VG settings in premature infants with RDS, using the open-lung strategy. According to the results, a specific set of numbers could not be recommended to achieve normocarbia. Following the trend of each patient and small adjustments according to the closely monitored pCO_2_ levels seems logical.

## Introduction

Despite the rising trend of non-invasive ventilation techniques, up to 50% of extremely preterm infants having respiratory distress syndrome (RDS) need to be intubated and mechanically ventilated. Prevention of repeated opening and closing of the alveoli, avoiding fluctuant and excessive tidal volumes are the fundamentals of the lung protective mechanical ventilation (1). Volume-targeted ventilation (VTV) strategies are increasingly used in the care of neonates and offer many advantages by avoiding disproportionate tidal volumes (2, 3). High-frequency oscillatory ventilation (HFOV) has been used over 30 years in newborn babies with severe respiratory failure (4, 5). HFOV with volume guarantee (HFOV-VG) is a promising new ventilatory mode for the treatment of respiratory failure in newborns. Theoretically, HFOV-VG is expected to result in less lung injury since it reduces fluctuations of high frequency tidal volume (VThf), reduces the number of out-of-target pCO_2_ values and provides fewer hypoxia attacks compared with HFOV (6, 7).

During HFOV-VG, the clinician can set a target VThf, and the ventilator will automatically adjust the amplitude pressure to supply the targeted VThf. Tight control of VThf and automatic adjustments in amplitude using HFOV-VG may be particularly useful when the respiratory mechanics change rapidly (6, 8). Previous studies demonstrated that during HFOV-VG, the VThf can vary from 1 s to another, but it is kept very close to the target VThf in the long term (9).

CO_2_ excretion during HFOV is defined by the diffusion coefficient of CO2 (DCO_2_) as an indicator of alveolar ventilation. Since DCO_2_ (ml^2^/s) is formulated by “*DCO*_2_=*f x VThf*^2^”, even small changes in VThf affect DCO_2_ more than changes in frequency (10–12). DCO_2_ has been considered an important parameter in the follow-up of CO_2_ elimination, however, its value providing normocapnia varies from patient to patient. Recently, weight-corrected DCO_2_ ([mL/kg]^2^/s) has been proposed to reduce inter-individual variability (7).

To ensure optimal benefit from the HFOV, the alveoli should be opened and kept open using optimal continuous distension pressures (13, 14). Though lung volume recruitment and appropriate tidal volume settings are considered important strategies for the success of HFOV-VG, optimal VThf parameters are not identified yet for preterm infants during the acute phase of RDS. This study's objective is to evaluate optimal VThf and DCO_2_ levels to achieve normocapnia during HFOV-VG using open-lung strategy in very premature infants with RDS.

## Materials and Methods

### Study Design, Patients, and Interventions

The retrospective observational study was carried out at the third level neonatal intensive care unit (NICU) at Dokuz Eylul University Hospital, between January 2014 and January 2019. The Ethical Committee of Dokuz Eylul University Faculty of Medicine approved the study protocol. Infants suffering from RDS between 23 and 32 weeks of gestation, who started HFOV-VG with lung recruitment maneuver within 72 h of life, were eligible for the study. Infants who had (i) congenital anomalies affecting the cardiopulmonary system or (ii) endotracheal tube leaks of over 40% or (iii) air leak syndromes or (iv) pulmonary hypertension were excluded.

All interventions were performed according to the unit's ventilation protocol by considering the infants' characteristics. Surfactant treatment was given according to the European Consensus Guidelines of that period. If surfactant was considered, 200 mg/kg (poractant alpha) was given to the first dose and repeated doses were given as 100 mg/kg (15, 16). The endotracheal tube diameter was selected according to the current NRP guidelines and the maximum tolerable tube leakage was 40% according to the operating principles of the ventilator and our unit's ventilation protocol (17). Suction was avoided unless clinically indicated. If suction was needed a closed system is preferred. All of these patients received fentanyl analgesia; strong sedatives and muscle relaxants were not used.

### Ventilation Strategies

We have been using HFOV-VG as elective or early-rescue ventilation mode for infants who were failing conventional ventilation or would benefit from HFOV according to the opinion of the attending clinician. In general, HFOV-VG ventilation has been started with the following reasons: higher VT need in conventional VG ventilation (> 6 ml/ kg), high peak positive pressure requirement (over 20 mbar), diffuse lung atelectasis requiring lung recruitment, or high FiO2 need (40%) despite proper PEEP and surfactant therapy.

All patients were treated with a ventilator (Dräger Babylog VN500) in HFOV-VG mode. An optimal volume strategy was applied in all infants. Depending on HFOV-VG starting time, MAPhf level was initiated with 8 or 2 mbar above the MAPhf in conventional mechanical ventilation. The MAPhf was increased with steps of 1 mbar every 2–3 min until a critical opening pressure, where oxygenation no longer improved, or the fraction of inspired oxygen (FiO_2_) was ≤ 0.30, to give an arterial oxygen saturation of 90–94%. Next, the MAPhf level was decreased by 1–2 mbar stepwise every 2–3 min to find the closing distending pressure. Finally, the lung was reopened again with the previously defined critical opening pressure and a MAPhf level 2 mbar above closing pressure was set, corresponding to optimal continuous distending pressure (18). The lung recruitment maneuver was performed under VG mode to allow amplitude fluctuations to obtain stable VThf levels. Recruitment was stopped in case of bradycardia (heart rate <100) or hypotension.

All of the infants received a frequency between 10 and 12 Hz and an oscillatory inspiratory/expiratory ratio of 1:1. The amplitude limit (Ampl _max_) was set at 10–15% above the average amplitude required to reach the target VThf. Subsequently, the set VThf was adjusted by the clinical team up or down in increments of 0.1–0.2 mL/kg if the pCO_2_ value was outside the target range. Capillary blood gases were assessed 30 min after the initiation of HFOV-VG and repeated at intervals of 4–6 h or more often as needed using a blood gas analyzer (ABLTM 700 Radiometer, Copenhagen, Denmark). The consecutive blood gases that belong to the period in which HFOV was applied continuously without interruption were selected for the study. The normocapnia was defined as pCO_2_ ranging from 40 to 55 mm Hg. Fractional inspired oxygen concentration (FiO_2_) was adjusted to obtain a SaO2 between 90 and 94% by a pulse oximeter.

### Data Acquisition and Analysis

The flow of gases in the airway and tidal volume was measured continuously using a hot wire anemometer positioned in the airway entrance while undertaking HFOV+VG. Critical parameters such as MAPhf (Paw), ΔPhf (swinging pressure around the mean Paw), VThf (ml), and DCO_2_ (mL^2^/s) were followed using VentView 2.n software (Draeger, Lubeck, Germany). The mean values of these parameters were assessed over 10 min before blood gas analysis was recorded. All calculations and correlations regarding VThf were performed according to the tidal volume normalized to body weight and given as mL/kg. Daily respiratory mechanics and blood gas analysis results and follow-up data were collected from the electronic and published patients' files.

### Statistics

The normality of data was determined by the Shapiro-Wilk test. According to the distribution pattern, continuous data were presented as mean ± standard deviation (SD) or median (25–75 percentile). The statistical significance of mean differences between two independent groups was tested using the independent *t*-test. To test the significance of the difference between the means or medians of parameters in three or more independent groups, one-way ANOVA or Kruskal Wallis tests were performed, respectively. The relationship between categorical variables was tested with chi-square analysis. Pearson's correlation analysis was performed to determine the correlations between selected parameters. The effect of multiple independent variables on pCO_2_ and optimal VThf levels was tested using the general linear model.

*Post-hoc* power analysis for differences of means was performed using an online statistical tool, “OpenEpi” (19). SPSS software was used for all statistical analyses (*IBM SPSS Statistics* for Windows, Version 24.0. Armonk, NY). Statistical significance was set at *p* < 0.05.

## Results

In total, 53 babies were included in the study between January 2014 and January 2019. Mean gestational age and birth weight of the infants were 26.3 ± 2.3 weeks and 882 ± 286 grams, respectively.

All of the infants received surfactant therapy in the delivery room or in the NICU according to the intubation time. Poractant alfa was instilled at 200 mg/kg for the first dose and then repeated at 100 mg/kg if needed according to RDS guidelines (15, 16). Delivery room intubation was performed for 38 infants. Median intubation time for the remaining patients was 10 h (min-max: 1–53 h). In 14 of these patients, a 12 Hz frequency was used according to the decision of the consultant neonatologist. The patient characteristics are shown in Table 1.

**Table 1:** Patient characteristics.

**Characteristic**	***n* = 53**
Gestational age (wk), mean ± SD	26.9 ± 2.4
Birth weight (g), mean ± SD	882 ± 286
Antenatal steroid, *n* (%)	30 (56.6)
Cesarean section *n* (%)	41 (77.4)
Male gender, *n* (%)	30 (56.6)
5' Apgar score, median (25–75 p)^*^	7 (5–8)
Intubation time (hour), median (25–75 p)	1 (1–1.5)
HFOV start time (hour), median (25–75 p)	8 (2–16)
Surfactant doses, median (25–75 p)	2 (1–3)
HFO duration (hour), (25–75 p)	49 (25–60)

* *Median (25–75 percentile)*.

Overall, the mean applied VThf per kg was 1.64 ± 0.25 mL/kg in the study sample. Of the 274 blood gases evaluated, 178 (65.2%) were in the normocarbic range, while the remaining were within the hypocarbic (53 blood gases, 19.4%) or hypercarbic (42 blood gases, 15.4%) range. Only nine patients needed VThf ≥2 mL/kg to overcome hypercarbia during follow-up, and none of these babies required VThf > 2.4 mL/kg and DCO_2_corr >50 [mL/kg]^2^/s to control hypercapnia. Four patients needed VThf settings smaller than 1.25 mL/kg for maintaining normocarbia. The patients that need very low VThf volumes were extremely preterm infants born at gestational age under 25 weeks.

**Figure 1:**
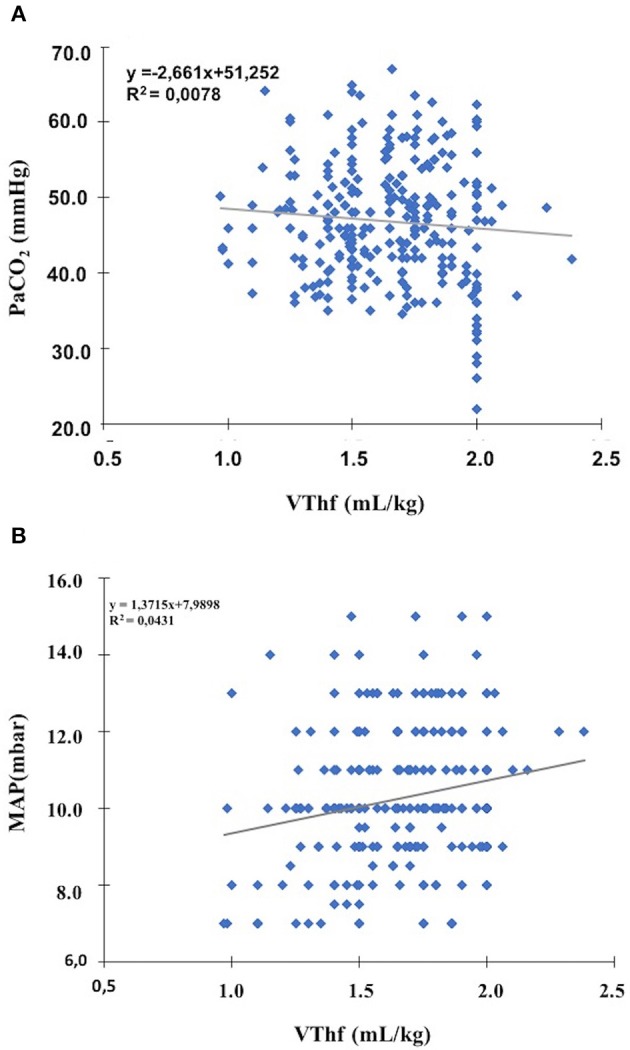
Pearson correlation analysis between VThf, pCO_2_, and MAPhf levels **(A)** no significant correlation between all VThf and pCO2 levels (Pearson coefficient = 0.01, *p* = 0.899) **(B)** a very weak, but significant correlation between optimal VThf and MAPhf levels (Pearson coefficient = 317, *p* < 0.001).

The mean VThf level yielding normocapnic blood gases was 1.61 ± 0.25 mL/kg. Mean VThf levels corresponding to these three pCO_2_ categories did not significantly differ between one or the other (Table 2). No significant correlation was found between VThf values and the corresponding pCO_2_ values during the follow-up period (Figure 1A). When the ventilatory settings corresponding to normocapnic blood gases were examined, there was a very weak but significant positive correlation between VThf and MAP values (0.319, *p* < 0.001) (Figure 1B).

**Table 2:** Ventilatory parameters corresponding to three pCO2 categories^*^.

**Parameter**	**Hypocarbia *n* = 53**	**Normocarbia *n* = 178**	**Hypercarbia *n* = 42**	***p***
Vt hf (mL/kg)^*^				
f 10 Hz	1.70 ± 0.24	1.65 ± 0.25**^#^**	1.68 ± 0.22	0.50
f 12 Hz	1.64 ± 0.27	1.50 ± 0.24**^#^**	1.60 ± 0.20	0.09
Amplitude (mbar)^**^				
f 10 Hz	15 (13–20)	17 (14–20)	18.7 (15–25)	0.119
f 12 Hz	15 (12–20)	16 (12–18)	15 (10.5–16)	0.823
DCO_2_ ml^2^/s^*^				
f 10 Hz	26.1 ± 17.2	27.2 ± 17.3	29.7 ± 14.4	0.724
f 12 Hz	16.1 ± 6.6	21.06 ± 13.5	32.1 ± 17.6	0.085
DCO_2corr_ [mL/kg]^2^/s^*^				
f 10 Hz	31.0 ± 8.2	29.4 ± 8.0	29.3 ± 6.3	0.599
f 12 Hz	32.9 ± 9.4	30.1 ± 7.4	35.4 ± 5.8	0.085
MAPhf (mbar)^**^				
f 10 Hz	10 (9–10)	10 (10–12)	10.5 (9.6–12)	<0.001
f 12 Hz	9 (8–10)	10 (8–11)	10.5 (9.6–12)	0.117
FiO2^*^				
f 10 Hz	31.5 ± 21.2	29.6 ± 11.0	32.4 ± 15.4	0.561
f 12 Hz	36.0 ± 17.5	30.2 ± 9.8	34.0 ± 8.0	0.113

* *Mean ± SD*.

** *Median (25–75 percentile)*.

# *Significant difference between two means p < 0.001*.

Subgroup analysis according to the frequency demonstrated that VThf levels to achieve normocarbia were significantly lower with 12 Hz frequency compared to 10 Hz frequency (1. 50 ± 0.24 vs. 1.65 ± 0.25 ml/ kg, *p* < 0.001) (Table 2).

Overall, the mean DCO_2_ level to obtain normocarbia was 25.67 ± 16.55 ml^2^/s and weight-corrected mean DCO_2_corr level providing normocarbia was calculated as 29.84 ± 7.88 [mL/kg]^2^/s (Table 2). There was no significant difference in DCO_2_corr levels between normocapnic, hypercapnic, or hypocapnic blood gases levels (*p* = 0,415) (Table 2). A significant correlation could not have been found between DCO_2_corr levels and pCO_2_ values.

To test the effect of multiple factors on optimal VThf values (ml/kg), birth weight, gestational age, frequency, and MAPhf levels were tested in a model using a general linear model. The results demonstrated that frequency and MAPhf levels were the main effectors of optimal VThf value (*p* = 0.02 and *p* = 0.003, respectively). Main ventilatory parameters were given as Supplementary Data (Table S1).

## Discussion

To our knowledge, this is the largest case series to evaluate HFOV-VG settings in premature infants with RDS. Results demonstrated that neither optimal VThf (mL/ kg) nor DCO_2_corr levels ([mL/kg]^2^/s) correlated with pCO_2_ levels. It was also confirmed that higher frequencies need lower delivered tidal volumes for adequate ventilation during HFOV-VG.

HFOV- VG mode makes it possible to maintain DCO_2_ and normocapnia while lowering VThf and increasing the frequency in an attempt to minimize lung injury (20, 21). In the present study, mean VThf levels corresponding normocapnic blood gases were around 1.50 cc mL /kg for 12 Hz, and 1.65 mL/ kg for 10 Hz. González-Pacheco et al. demonstrated that adequate VThf was 1.46 mL/kg for ELBW infants and 1.57 mL/kg for infants weighing 1,000–2,000 g using higher frequencies up to 17 Hz (21). In our study a VThf level over 2.4 mL/kg was not required. Consistent with these results, Belteki et al. demonstrated that VThf or DCO_2_ have poor correlation with CO_2_ levels but a volume of >2.5 mL/kg VThf is rarely needed (7). Parallel to our results, Zimova-Herknerova et al. showed that the median delivered normocapnic VThf during HFOV was 1.67 mL/kg in a heterogeneous group of newborns ventilated by HFOV at any time during their hospital stay (12). Few studies support higher VThf requirement during HFOV varying between 1.75–1.90 mL/kg using a constant frequency of 10 Hz (6, 22). Besides the frequency, gestational age, HFOV starting time, practicing open lung strategies, and severity of lung disease are possible determiners of VThf need. Our population was composed of a near- homogeneous group of patients suffering from RDS. We started HFOV as an early-rescue mode rather than rescue HFOV and median starting time (8 h) is indicating the initial phase of RDS rather than the other problems, such as hemodynamically significant PDA, pulmonary edema, VILI, chronic lung disease etc.

Although VThf is considered a key element for minute ventilation in HFOV, our study did not demonstrate a correlation between VThf and pCO_2_ levels. Even the same patients had out-of-target pCO_2_ levels with the same VThf levels. As a possible reason, allowing spontaneous breathing without the use of heavy sedatives or muscle relaxants may have affected the gas exchange in these infants in different ways. The narrow range of VThf values applied in this study may explain the similarity of mean VThf values corresponding to normocapnic and hypo or hypercarbic blood gases. If we used higher VT values above 2 ml/kg, we might have seen the expected relationship between hypocarbia and high VT. Furthermore, we considered a relative narrow pCO_2_ range between 40 and 55 mmHg as normocarbia. Despite this, 65% of the blood gases were normocarbic. If a larger range (5–8 kpa = 37.5–60 mmHg) had been considered as in the other studies (9) the rate of normocarbic blood gases would have been increased to 87.6%, a successful rate considering the high incidence of hypocapnia during HFOV.

Currently, a VThf requirement according to the patient's characteristics such as birth weight and disease severity is not as well-known as in conventional VG ventilation. However, a weak but significant correlation between MAPhf and optimal VThf levels in this study may indicate the need for higher VThf levels in infants with more severe lung disease or inadequate lung recruitment. Patients who responded well to recruitment probably needed lower MAPhf and VThf levels due to decreased physiologic dead space and shunt, similar to ARDS patients (23). However, there is no strong evidence to support this assumption.

In our study, the DCO_2_ level was highly variable according to the birth weight of the infants. Then we considered the calculation of the DCO_2_corr levels as previously recommended (7). Although DCO_2_ is considered the best predictor of CO_2_ elimination during HFOV, our study could not demonstrate a significant correlation between optimal VThf and DCO_2_ levels. Targeting a weight-corrected DCO_2_corr achieved more static levels; however, this adjustment does not change the results because there was not a significant correlation between DCO_2_corr and VThf levels.

This study has several limitations. The most important limitation was its retrospective design. This patient group was selected since it was a relatively homogenous population composed of very preterm infants and started HFOV-VG because of severe RDS in the first 72 h of life. Although the selected study population is not heterogeneous, these results may not be generalized for all premature infants with RDS. Although the retrospective design and longer study period may be questionable, over the 5 years, our unit's RDS and HFOV-VG protocols have not been modified too much. The lack of continuous CO_2_ monitoring during the infants' course is another important limitation. This restriction may lead to an incomplete representation of the real-time relationship between evaluated parameters. Ideally, instant monitoring of pCO_2_ and ventilation parameters prospectively in a homogeneous patient group will allow a clear understanding of the relationship between them.

In summary, results could not allow making any recommendation on an optimal starting value for VThf. Optimal levels are dynamic and vary according to the instantaneous features of individuals. Therefore, it is unrealistic to recommend a general VThf value to all patients. The needs of each patient should be monitored within itself and the settings should be titrated according to close pCO_2_ monitoring.

## Data Availability Statement

All datasets generated for this study are included in the article/Supplementary Material.

## Ethics Statement

The studies involving human participants were reviewed and approved by Dokuz Eylul University Clinical Researches Ethical Commity. Written informed consent from the participants' legal guardian/next of kin was not required to participate in this study in accordance with the national legislation and the institutional requirements.

## Author Contributions

FT, BD, and HO contributed to the conception and design of the study. ND, BI, MC, and BD organized the database. FT performed the statistical analysis and wrote the manuscript. BI wrote the first draft of the manuscript. All authors contributed to manuscript revision, read and approved the submitted version.

### Conflict of Interest

The authors declare that the research was conducted in the absence of any commercial or financial relationships that could be construed as a potential conflict of interest.

## Supplementary Material

Table S1 Main ventilatory parameters.
